# Identification and characterization of early Fusarium wilt responsive mRNAs and long non-coding RNAs in banana root using high-throughput sequencing

**DOI:** 10.1038/s41598-021-95832-8

**Published:** 2021-08-11

**Authors:** Chunzhen Cheng, Fan Liu, Na Tian, Raphael Anue Mensah, Xueli Sun, Jiapeng Liu, Junwei Wu, Bin Wang, Dan Li, Zhongxiong Lai

**Affiliations:** 1 Institute of Horticultural Biotechnology, Fujian Agriculture and Forestry University, Fuzhou, 350002 China; 2 College of Horticulture, Shanxi Agricultural University, Taigu, 030801 China

**Keywords:** Biotechnology, Molecular biology

## Abstract

Fusarium wilt disease, caused by *Fusarium oxysporum* f.sp. *cubense* (*Foc*), has been recognized as the most devastating disease to banana. The regulatory role of long non-coding RNAs (lncRNAs) in plant defense has been verified in many plant species. However, the understanding of their role during early *Foc*TR4 (*Foc* tropical race 4) infection stage is very limited. In this study, lncRNA sequencing was used to reveal banana root transcriptome profile changes during early *Foc*TR4 infection stages. Quantitative real time PCR (qRT-PCR) was performed to confirm the expression of eight differentially expressed (DE) lncRNAs (DELs) and their predicted target genes (DETs), and three DE genes (DEGs). Totally, 12,109 lncRNAs, 36,519 mRNAs and 2642 novel genes were obtained, of which 1398 (including 78 DELs, 1220 DE known genes and 100 DE novel genes) were identified as *Foc*TR4 responsive DE transcripts. Gene function analysis revealed that most DEGs were involved in biosynthesis of secondary metabolites, plant–pathogen interaction, plant hormone signal transduction, phenylalanine metabolism, phenylpropanoid biosynthesis, alpha-linolenic acid metabolism and so on. Coincidently, many DETs have been identified as DEGs in previous transcriptome studies. Moreover, many DETs were found to be involved in ribosome, oxidative phosphorylation, lipoic acid metabolism, ubiquitin mediated proteolysis, N-glycan biosynthesis, protein processing in endoplasmic reticulum and DNA damage response pathways. QRT-PCR result showed the expression patterns of the selected transcripts were mostly consistent with our lncRNA sequencing data. Our present study showed the regulatory role of lncRNAs on known biotic and abiotic stress responsive genes and some new-found *Foc*TR4 responsive genes, which can provide new insights into *Foc*TR4-induced changes in the banana root transcriptome during the early pathogen infection stage.

## Introduction

Banana (*Musa* spp.) is not only one of the most important and popular fruit worldwide but also an important crop in many tropical and subtropical countries^1,2^. As a popular fruit, its global trade amount ranked first among all the fruits^1,2^. As a crop, it serves as staple food for millions of people^3–5^. Cultivated banana varieties are generally seedless and vegetatively propagated hybrids domesticated and originally derived from of the wild diploid *M. acuminata* and *M. balbisiana*^4,6–8^. Currently, more than 90% of commercial export dessert bananas are derived from somaclones of Cavendish^4^, whose flavor is superb however has a relatively low disease resistance^1^. Thus, cultivated banana often suffers yield losses from different kinds of diseases caused by bacterial, fungal and viral pathogens, among which the Fusarium wilt disease (FW, also known as Panama disease) caused by soil-borne fungi *Fusarium oxysporum* f.sp. *cubense* (*Foc*), has been identified as the most devastating. The chlamydospore of *Foc* can survive in the soil without its banana host or in barren soil for 30 years, making the complete control of this disease through conventional agricultural measures such as rotation nearly impossible^9^. Moreover, this disease spreads rapidly through several different means, greatly weakening effects of the prevention methods^10^. What’s worse, the host range of *Foc*s in banana especially *Foc* tropical race 4 (*Foc*TR4) is very wide, this strain can infect almost all banana varieties at all growing stages in all banana growing regions and induce wilt symptoms eventually leading to plant death^9^. Although enormous attempts, including biological, chemical and agricultural control methods have been tried, up to now, it is still impossible to completely settle this matter. Thus, breeding FW resistant banana cultivars is one important fundamental and practical way to mitigate the problem. As conventional crossbreeding tend to be time consuming, other methods for obtaining resistant cultivars should also be employed in the quest to fight this devastating pathogen. Transgenic breeding could be used to meet the urgent need of creating more banana varieties that are resistant to *Foc* in relatively short time. Nevertheless, the candidate resistance transcripts are very limited. Therefore, the mining of the candidate transcripts related to FW resistance stands in the breach^10^. High throughput sequencing method has been applied by scientists to compare the mRNA changes of one banana variety at different infection time points after *Foc* 1 or *Foc*TR4 infection^11,12^ or both^13^, and two banana varieties (one is tolerant to *Foc*TR4 and the other is susceptible) to *Foc*TR4 infection^10^ for the exploration of candidate FW resistant genes. These transcriptomic analysis provided insights into the molecular mechanism associated with banana-*Foc* interaction, and genes involved in phenylalanine metabolism, phenylpropanoid biosynthesis, alpha-linolenic acid metabolism, phytohormone biosynthesis and signaling, cell wall lignification and so on were identified to contribute to the FW resistance of banana.

Long noncoding RNAs (lncRNAs), a class of transcripts of more than 200 nucleotides but with no apparent coding sequence (CDS) or open reading frame (ORF)^14–16^, were once considered as expression noise of protein coding mRNA. They were often missed during the transcriptome analysis in previous studies. Recently, however, accumulating evidences showed that lncRNAs play important regulatory roles in many essential biological processes and various biotic and abiotic stress responses^17–19^. Their regulatory role in flower development^20–25^, fertility^26^, photomorphogenesis^27^, sexual production^28^, seed germination, development and growth^29–31^, yield^32^, fruit development and ripening^33–37^, biotic and abiotic stress responses^38–49^, and other important developmental processes^50–55^ have attracted great attentions from researchers.

The role of lncRNAs during plant–pathogen interactions has also been uncovered in many plant species. For example: in wheat, Zhang et al.^56^ identified four differentially expressed (DE) lncRNAs that regulating the expression of plant defense related genes in response to *Puccinia striiformis* f. sp. *Tritici* (*Pst*) infection. Zhang et al.^57^ investigated the *Triticum aestivum* transcriptome changes in response to *Pst* and *Blumeria graminis* f. sp. *tritici* (*Bgt*) infection, identified 254 *Bgt* responsive and 52 *Pst* responsive long intergenic ncRNAs (LincRNAs). In *Arabidopsis thaliana*, 35 disease resistance related *F. oxysporum*-induced lncRNAs were identified^40^. In tomato, a lncRNA Slylnc0195 was identified to function during tomato-TYLCV interaction by competing with miR166 to maintain the expression of its target genes^14^. lncRNA16397 could also induce the expression of its target gene (*SlGRX*) to reduce the damage caused by *Phytophthora infestans* infection^58^. Jiang et al.^59^ revealed the role of tomato lncRNA23468 in promoting *P. infestans* resistance by functioning as an eTM (endogenous microRNA target mimic) of miR482b. The overexpression of tomato lncRNA *SlLNR1* could enhance host resistance to tomato yellow leaf curl virus (TYLCV)^60^. In *Brassica napus*, Joshi et al.^61^ found one *Sclerotinia sclerotiorum* responsive lncRNA, TCONS_00000966, showed 90% overlap of a *defensin* gene, suggesting its involvement in *B. napus*–*S. sclerotiorum* infection. In rice, Jain et al.^62^ identified 2000 lncRNAs in mock and *Magnaporthae oryzae* inoculated rice samples and pointed out that many lincRNA candidates obtained from resistant rice line contributed to its disease resistance. Zhang et al.^63^ compared the expression of lncRNAs in two cotton species (one is resistant to *Verticillium dahliae* and the other susceptible to *V. dahlia*), and found that the silencing of *GhlncNAT-ANX2* and *GhlncNAT-RLP7* could enhance disease resistance by increasing the expression of *LOX1* and *LOX2*. Yao et al.^64^ reported that lncRNA contributed a lot to the susceptibility of sea-island cotton recombinant inbred lines to *Fusarium oxysporum* f. sp. vas *infectum* infection by regulating the expression of genes involved in disease resistance-related pathways. Cao et al.^65^ identified 748 DELs in response to phytoplasma infection in *Paulownia tomentosa*. The target genes of these DELs were mainly involved in lignin biosynthesis, plant pathogen interaction and plant hormone signaling and so on, suggesting that lncRNA play important role in Paulownia–phytoplasmas interaction, at least partially, by regulating the plant defense pathways^65^.

For banana, lncRNAs have been studied in relation to genome-wide lncRNA identification^1,2,4,66^. Muthusamy et al.^5^ compared the drought stress responsive lncRNAs between drought tolerant and susceptible cultivars, and identified > 8000 drought responsive lncRNAs. In our previous study, we identified 12,462 lncRNA from cold resistant wild banana and revealed their possible roles its cold resistance properties^67^. Li et al.^68^ reported the lncRNA expression profile changes of FW resistant ‘Nongke No.1’ and susceptible ‘Brazil’ in response to *Foc*TR4 infection at 27 h post inoculation (hpi) and 51 hpi, and found that some DELs were related to plant–pathogen interaction and phytohormone signal transduction. But the method they used for *Foc*TR4 inoculation, i.e. artificially damaging or uncovering the root epidermis and then covering the wound with freshly prepared *Foc*TR4 block, might result in the missing of the DELs that function in the early stages (such as the root attachment stage and the stage that *Foc*TR4 began to infect into the root epidermis) and the infiltration of wound-responsive lncRNAs. Therefore, in the present study, to reveal the role of lncRNAs in banana in response to *Foc*TR4 infection in the early stages, lncRNA sequencing was applied to reveal the changes in lncRNA profiles in roots of *Foc*TR4-inoculated ‘Tianbaojiao’ banana (*Musa acuminata* cv. Tianbaojiao), a FW susceptible banana cultivar, in the early *Foc*TR4 infection stages (5 hpi, 10 hpi and 25 hpi)^69^. Our findings can provide new insights into the *Foc*TR4-induced changes in the banana transcriptome and will further facilitate the understanding of banana response to FW as well as the pathogenesis of *Foc*TR4 in the early stages.

## Materials and methods

### Plant materials and FocTR4 inoculation

The ‘Tianbaojiao’ banana (*Musa acuminata* cv. Tianbaojiao) used in this study is a FW susceptible banana cultivar that has been grown in Tianbao town, Zhangzhou city, Fujian province of China for more than 700 hundred years and is one of the main banana cultivar there^69^. Seedlings were provided by Institute of Horticultural Biotechnology, Fujian Agriculture and Forestry University, Fuzhou, China. GFP-labeled *Foc*TR4 strain was graciously provided by College of Plant Protection, Fujian Agriculture and Forestry University (Fuzhou, China) and was kept in our lab. Banana seedlings with five leaves and healthy root system were cultured in modified Hoagland solution for about 1 week for hydroponic cultivation adaptation^69^. The *Foc*TR4 spore solution was prepared according to our previous study^69^. Seedlings were treated with this spore solution with final concentration of 5 × 10^6^ chlamydospores / ml. Samples were divided into 4 groups, i.e. Root_CK (control), Root_5H (chlamydospores began to attach to the root), Root_10H (chlamydospores infected into root samples and chlamydospores began to germinate and develop into hyphae) and Root_25H (*Foc*TR4 began to infect into the vascular tissues), and inoculated with *Foc*TR4 for 0 h, 5 h, 10 h and 25 h^69^, respectively. To reduce the influence of culture time on gene expression, root samples of three plants from each treatment group were harvested simultaneously. Then, root samples were precooled in liquid nitrogen followed by storage in freezer at − 80 °C for further use.

### Total RNA isolation, RNA library construction, and high-throughput sequencing

Total RNA was isolated from each banana root sample using Trizol Reagent (Invitrogen, Carlsbad, CA, USA) and was treated with DNase I (RNase-free) to remove DNA. RNA quality and quantity were checked using 1% agarose gel electrophoresis, NanoPhotometer spectrophotometer (Implen, CA, USA) and Bioanalyzer 2100 system (Agilent Technologies, CA, USA). High quality root RNA from the three banana seedlings of the same group were equal-weighted mixed. rRNA was then removed from total RNA by treatment with Epicentre Ribo-zero rRNA Removal Kit (Epicentre, USA). 3 μg retrieved RNA was used for sequencing library construction using the rRNA-depleted RNA by NEBNext Ultra Directional RNA Library Prep Kit for Illumina (NEB, USA) according to the manufacturer manual. Then, cDNA products were purified using AMPure XP system. After library quality assessment on the Bioanalyzer 2100 system (Agilent Technologies, CA, USA), cDNA libraries were sequenced on Illumina HiSeq 4000 platform at Beijing Novogene Bioinformatics Technology Co., Ltd. to generate 150 bp pair-end reads.

### Read mapping and transcriptome assembling

After removing reads containing adapters, poly-N, low-quality and shorter reads from the raw data, the remaining clean data were mapped to the *Musa acuminata* genome (ftp://ftp.ensemblgenomes.org/pub/plants/release-18/gtf/musa_acuminata) using TopHat v2.0.9^70^ and assembled using Cufflinks 2.1.1^71^. Assembled transcripts longer than 200 bp were kept for further analysis.

### Identification of lncRNAs, DEGs and DELs

CPC (0.9-r2) and Pfam-scan (v1.3) were applied for the screening of candidate noncoding RNAs^25^. A transcript was deemed to have no protein-coding capacity if it had CPC < 0 and without hit in Pfam at − E^0.001^. Noncoding transcripts with FPKM > 0.5 were then selected out as the candidate lncRNAs. The lncRNAs were classified into several categories according to their genomic location and previous description by Roberts et al.^72^. DEGseq package was used to identify the DEGs and DELs with corrected *p* values < 0.05 and log_2_(fold change) > 1 or < − 1^73^.

### Function prediction of DE lncRNA

The 100 k upstream and downstream coding genes were considered as *cis*-acting targets of lncRNAs^74^. For the DEL function prediction, the GO enrichment analysis of their target genes was analyzed using GOseq^75^, the KEGG enrichment analysis was performed using KOBAS according to Liu et al.^67^. For GO and KEGG enrichment analysis, corrected *p* value < 0.05 was used as criteria.

### Quantitative real‑time polymerase chain reaction (qRT‑PCR) analysis

Total RNAs were reverse transcribed into cDNA using PrimeScript RT Reagent (Prefect Real Time) Kit (Takara, Japan) for qRT-PCR analysis. The qRT-PCR was performed on a LightCycler 480 (Roche) as described by Liu et al.^67^ using *GAPDH* (*Glyceraldehyde-3-phosphate dehydrogenase*) and *EIF5A-2* (*Eukaryotic initiation factor 5A-2*) as the endogenous controls^76^. The expression of *WRKY40* (GSMUA_Achr11G08290_001), *TIFY 5A* (GSMUA_Achr7G01230_001) and *Cytochrome P450 93A1* (*P450 93A1*, GSMUA_Achr9G24040_001), TCONS_00083452 and its predicted target gene *MYB108* (GSMUA_Achr11G04930_001), TCONS_00363647 and its predicted target gene *Ethylene-responsive transcription factor ERF071* (*ERF071*, GSMUA_AchrUn_randomG27100_001), TCONS_00041355 and its predicted target *Tyrosine/DOPA decarboxylase 2* (*DOPA2*, GSMUA_Achr10G28740_001), TCONS_00093014 and its target gene *light harvesting chlorophyll a–b binding protein 6A* (*LHCB 6A*, GSMUA_Achr2G06350_001), TCONS_00214268 and its predicted target gene *tubulin beta-7 chain like gene* (*TUBB7*, GSMUA_Achr5G12310_001), TCONS_00334460 and its predicted target gene *geraniol 8-hydroxylase-like* (*G8H*, GSMUA_Achr9G24020_001), TCONS_00152125 and its predicted target gene *cytokinin hydroxylase-like* (*CTH*, GSMUA_Achr4G04400_001), TCONS_00334460 and its predicted target gene *Plastocyanin* (*PC*, GSMUA_Achr9G24120_001) was determined and their expression levels were quantified according to Zhou et al.^77^. Primers were designed using the Primer3 software (http://bioinfo.ut.ee/primer3-0.4.0/), and are listed in Supplementary Table S1.

### Statement

We confirm that the use of materials in the present study complies with the local guidelines and legislation.

## Results

### LncRNA sequencing and identification of genes and lncRNAs

**Figure 1:**
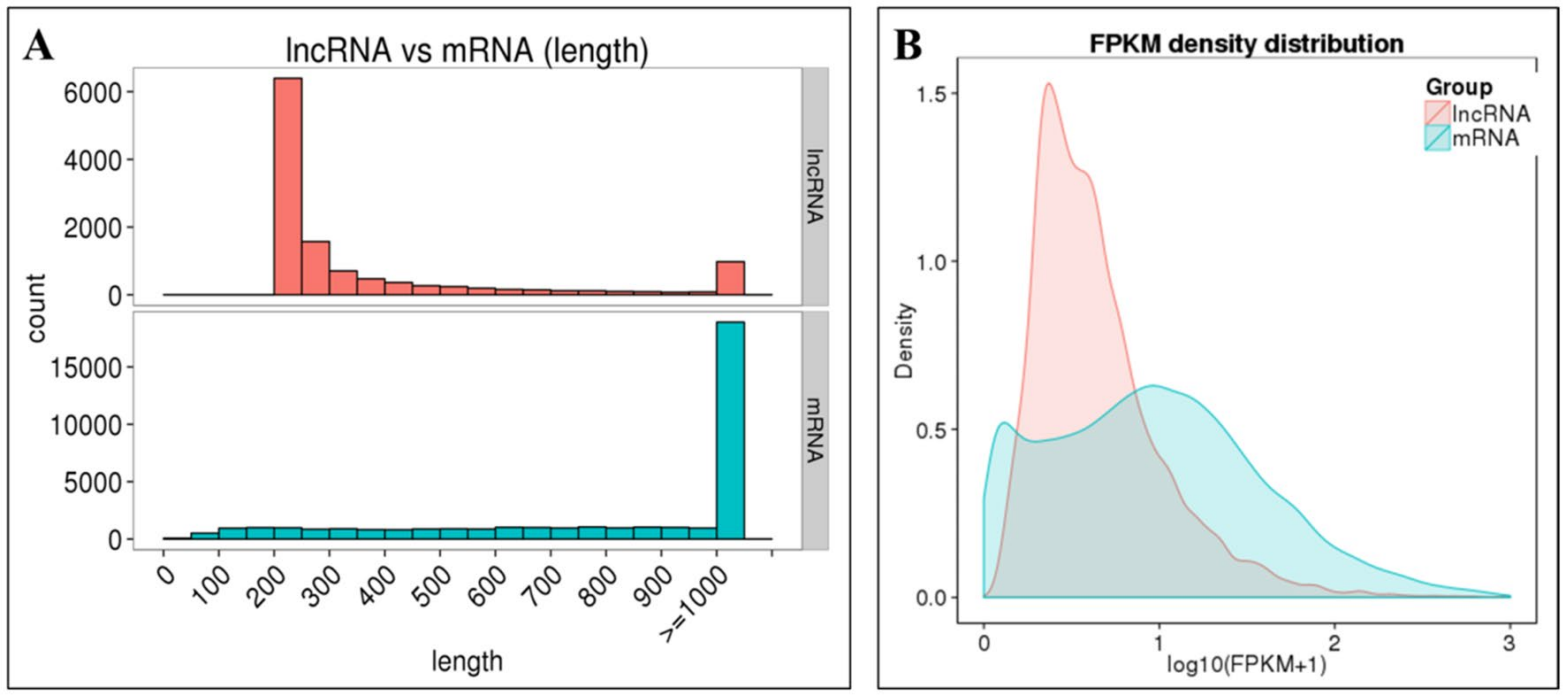
Length (**A**) and density (**B**) distribution result of the lncRNA and mRNAs identified in this study. (**A**) LncRNAs with length from 200 to 300 bp accounted for the largest part. (**B**) The lncRNA’s expression diversity was higher than mRNA, while their expression frequency was significantly lower than mRNA. The expression frequency was shown as log_10_(FPKM + 1). FPKM: fragments per kilobase million.

**Figure 2:**
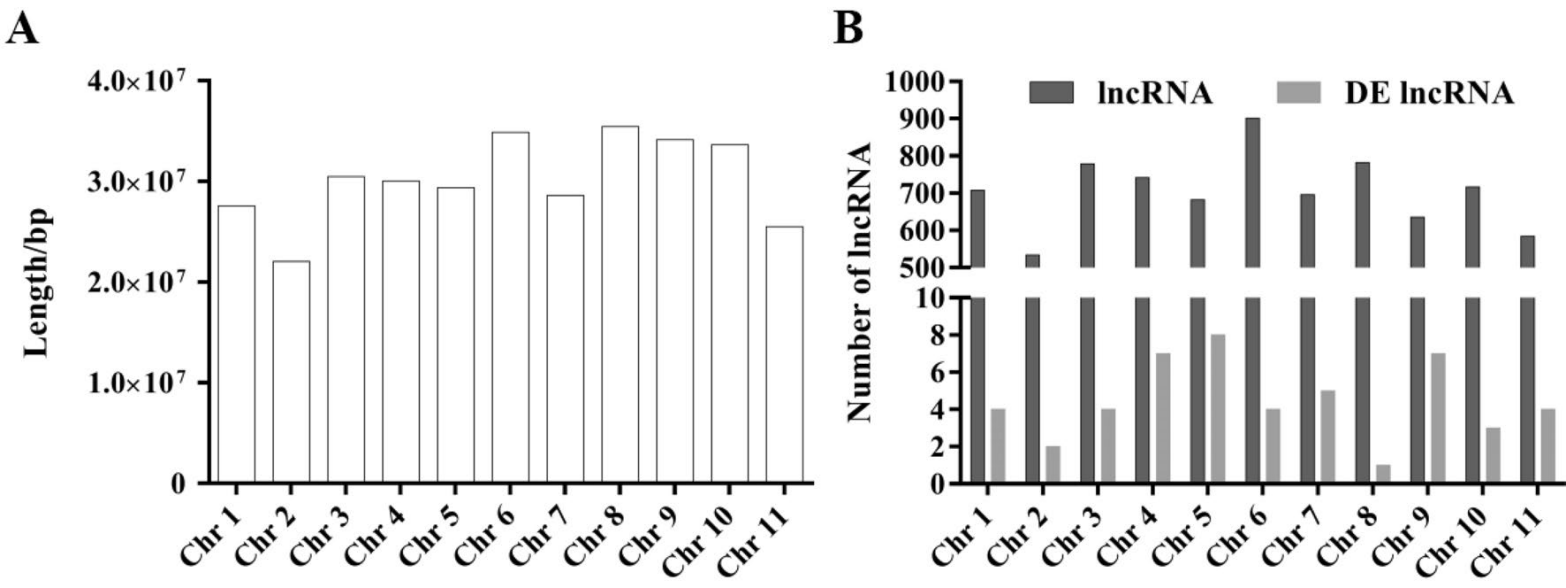
Chromosome length (**A**) and the distribution of long noncoding RNAs (lncRNAs) and chromosome location of the identified DE lncRNAs (**B**). DE: differentially expressed, Chr: chromosome, bp: base pair.

Four cDNA libraries (Root_CK, Root_5H, Root_10H and Root_25H) were sequenced using the Illumina HiSeq 4000 platform. For each library, more than 12.4 Gb clean bases with high Q20 and Q30 were obtained (Table 1). Totally, 96,826,792, 104,242,108, 84,492,752 and 100,085,710 raw reads, and corresponding 94,556,176, 102,114,506, 82,724,362 and 98,333,734 clean reads were gained from Root_CK, Root_5H, Root_10H and Root_25H library, respectively. 50.6–58.58% of clean reads could be mapped to the *M. acuminata* genome, and 49.29–57.38% of them were uniquely mapped reads. More than 60% of the mapped reads were mapped to protein coding genes (Table 1). In total, 36,519 known mRNAs, 2642 novel genes and 12,109 lncRNAs were identified. The expression diversity of lncRNAs was higher than mRNA, while their expression frequency was significantly lower than mRNA (Fig. 1), which was consistent with the result in *Miscanthus lutarioriparius*^53^. Chromosome distribution analysis revealed uneven lncRNAs distribution in the banana genome with the largest amount of lncRNAs identified in chromosome 6, followed by chromosome 8, 3, 4, 10 and 1 (> 700 lncRNAs). The least amounts of lncRNAs were identified in chromosome 2, which is the shortest chromosome of banana (Fig. 2). According to the location of lncRNAs in the banana genome, we identified 1686 intronic lncRNAs, 9365 lincRNAs and 1058 antisense lncRNAs. 11,655, 11,455, 11,675 and 11,752 known genes, 2155, 2310, 2208 and 2249 novel genes, and 7129, 7503, 7625 and 7851 lncRNAs were identified in Root_CK, Root_5H, Root_10H and Root_25H, respectively (Table 2, Supplementary Table S2).

**Table 1:** Sequencing data and mapping information of the four libraries used in this study.

	Root_CK	Root_5H	Root_10H	Root_25H
Raw reads	96,826,792	104,242,108	84,492,752	100,085,710
Clean reads	94,556,176	102,114,506	82,724,362	98,333,734
Clean bases	14.18G	15.32G	12.41G	14.75G
Q20 (%)	96.84	97.24	96.69	96.91
Q30 (%)	91.83	92.68	91.56	91.95
GC content (%)	54.32	50.80	52.89	53.54
Mapped reads	47,843,117	59,819,742	44,011,486	49,852,043
Unique mapped reads	46,607,613	58,591,552	42,829,452	48,722,627
miRNA	1671 (0.01%)	1612 (0.01%)	1460 (0.01%)	1779 (0.01%)
nontranslating_CDS	1209 (0.00%)	2212 (0.01%)	1536 (0.01%)	1444 (0.01%)
protein_coding	14,981,943 (60.28%)	21,248,489 (69.23%)	15,232,218 (66.33%)	15,765,821 (60.86%)
tRNA	1264 (0.01%)	1793 (0.01%)	1341 (0.01%)	1122 (0.00%)
Others	9,869,766 (39.71%)	9,437,438 (30.75%)	7,726,480 (33.65%)	10,136,075 (39.13%)

**Table 2:** The identified known genes, novel genes and lncRNAs in the four banana root groups through lncRNA sequencing.

Groups	Known genes		Novel genes		lncRNAs		lincRNAs		Intronic lncRNAs		Antisense lncRNAs	
	All	DE	All	DE	All	DE	All	DE	All	DE	All	DE
Root_CK	11,655	–	2155	–	7129	–	5551	–	927	–	651	–
Root_5H	11,455	1117	2310	87	7503	53	5933	39	878	5	692	9
Root_10H	11,675	240	2208	24	7625	36	6071	25	855	2	699	9
Root_25H	11,752	125	2249	17	7851	12	6198	5	966	0	687	7
Total	31,601	1220	2642	100	12,109	78	9365	57	1686	5	1058	16

DE: differentially expressed.

### Identification of DEGs and DE lncRNAs (DELs)

**Figure 3:**
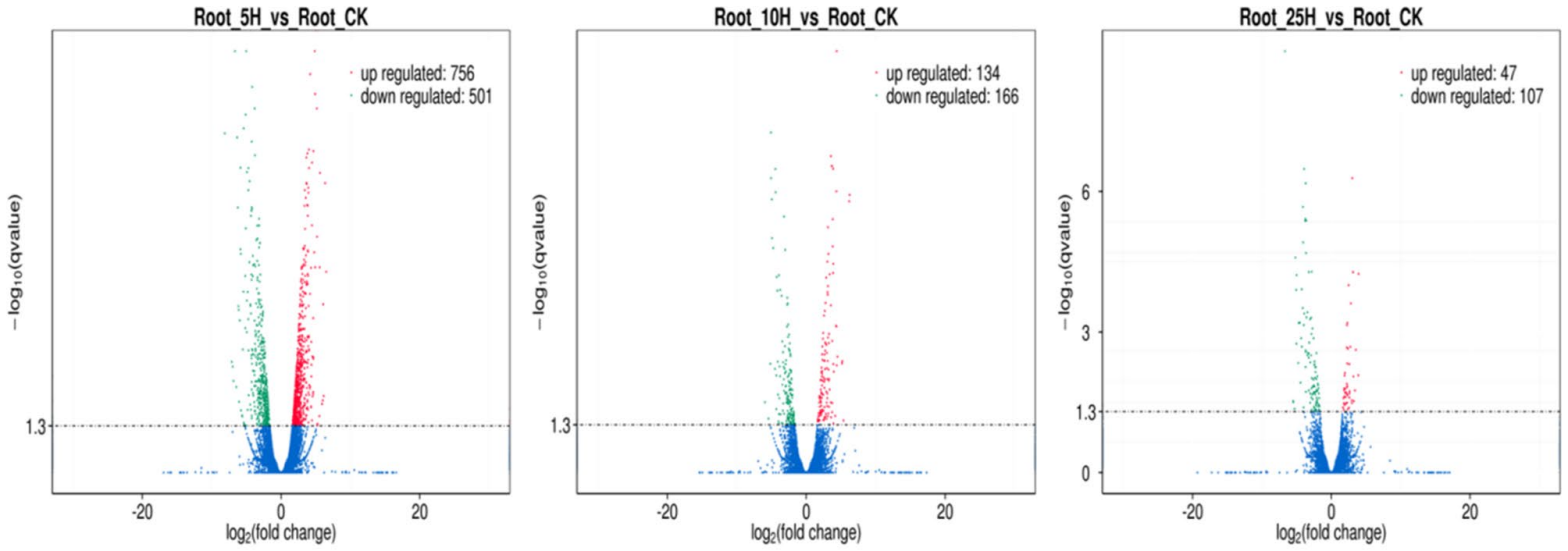
The volcano figures for the DE transcripts in the three comparisons. ‘Up regulated’ represents that the expression of the transcript in *Foc*TR4 treated sample is higher than the control, ‘down regulated’ represents that the expression of the transcript in *Foc*TR4 treated sample is lower than the control. The criteria used for screening the DE transcript is: corrected *p* values < 0.05 and log_2_(fold change) > 1 or < − 1.

**Figure 4:**
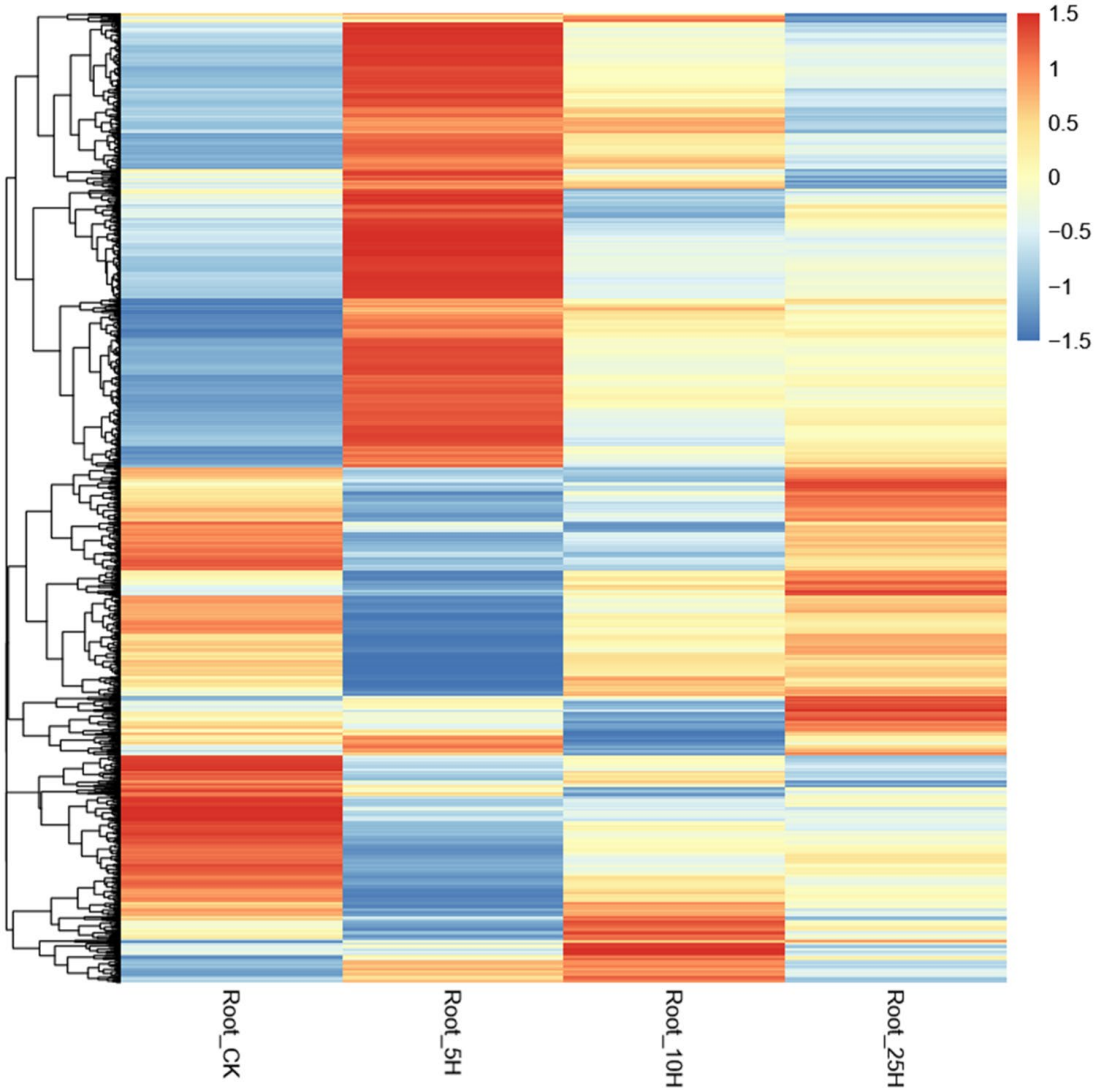
Heatmap for the expression of the identified DE transcripts in the four banana root lncRNA libraries clustered by log_10_ (FPKM + 1) value. DE: differentially expressed. FPKM: fragments per kilobase million. Root_CK, Root_5H, Root_10H and Root_25H is for the lncRNA library that roots were inoculated with *Foc*TR4 for 0 h, 5 h, 10 h and 25 h, respectively.

DEGs and DELs were identified by respectively comparing the RPKM of the transcripts in Root_5H, Root_10H and Root_25H with Root_CK using corrected *p* values < 0.05 and log_2_(fold change) > 1 or < − 1 (Comparison 1: Root_5H vs Root_CK, Comparison 2: Root_10H vs Root_CK; Comparison 3: Root_25H vs Root_CK) as the criteria. Totally, 1398 DE transcripts (including 1220 known genes, 100 novel genes and 78 lncRNAs) were identified (Supplementary Table S3), including 1257 DE transcripts (756 up-regulated and 501 down-regulated) in Comparison 1, 300 DE transcripts (134 up-regulated and 166 down-regulated) in Comparison 2, and 154 DE transcripts (47 up-regulated and 107 down-regulated) in Comparison 3 (Figs. 3, 4).

Notably, 48 DE transcripts, including 37 DE known genes, 6 DE novel genes and 5 DELs (Supplementary Table S4), were identified in all three comparisons. Notably, five genes encoding ethylene-responsive transcription factors (ERFs) were identified as DEGs (Supplementary Table S4), which again indicate that they function in response to *Foc*TR4 infection as described in previous studies^50,68^.

### Functional analysis of DEGs

**Figure 5:**
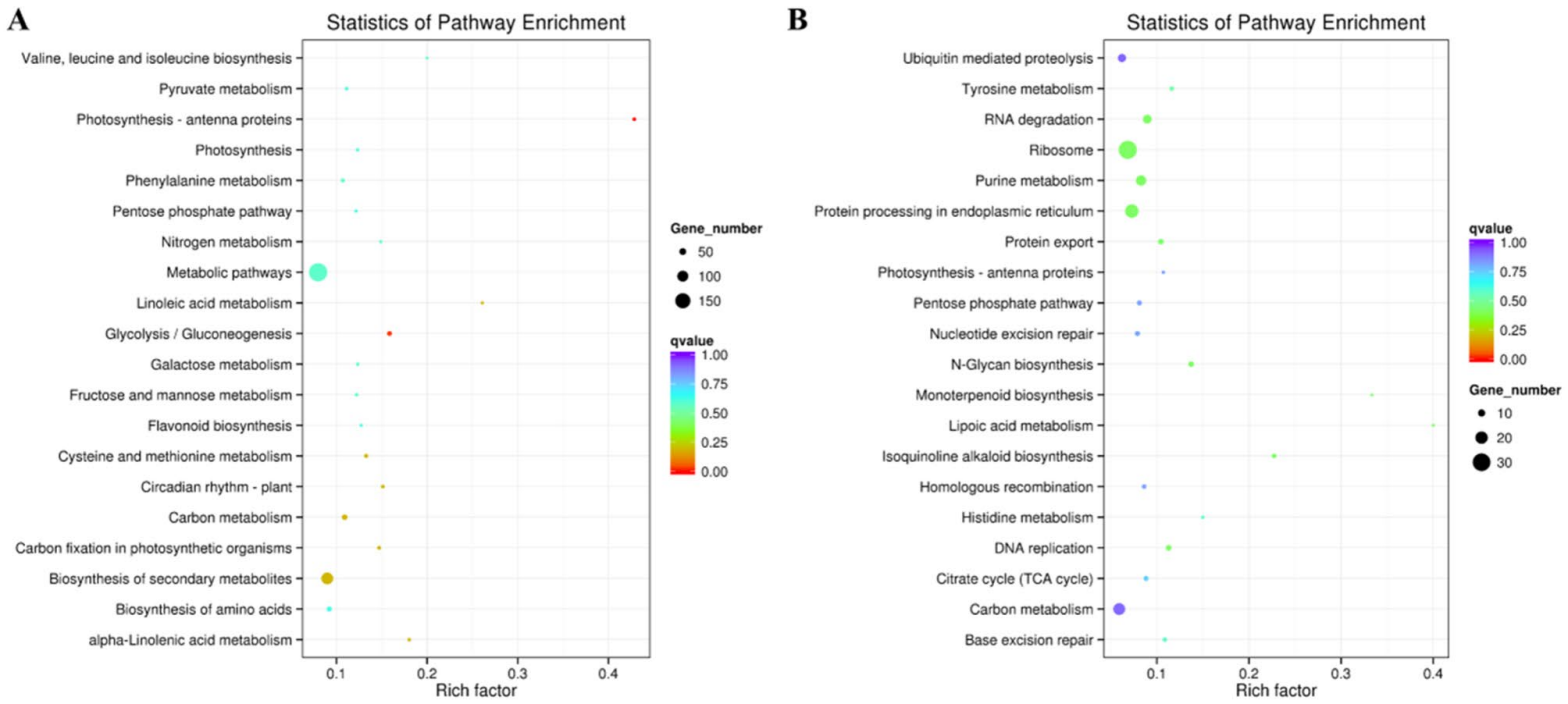
Statistics results of pathway enrichment of DEGs (**A**) and DETs (**B**). DEG: differentially expressed gene; DET: predicted target gene of the differentially expressed lncRNA. The color and the size of the circle represents the q-value and the number of DEGs or DETs enriched in the pathway, respectively. The rich factor represents the ratio between the number of enriched DEGs or DETs in a pathway and the number of annotated genes, the higher the rich factor is, the higher degree of enrichment.

**Figure 6:**
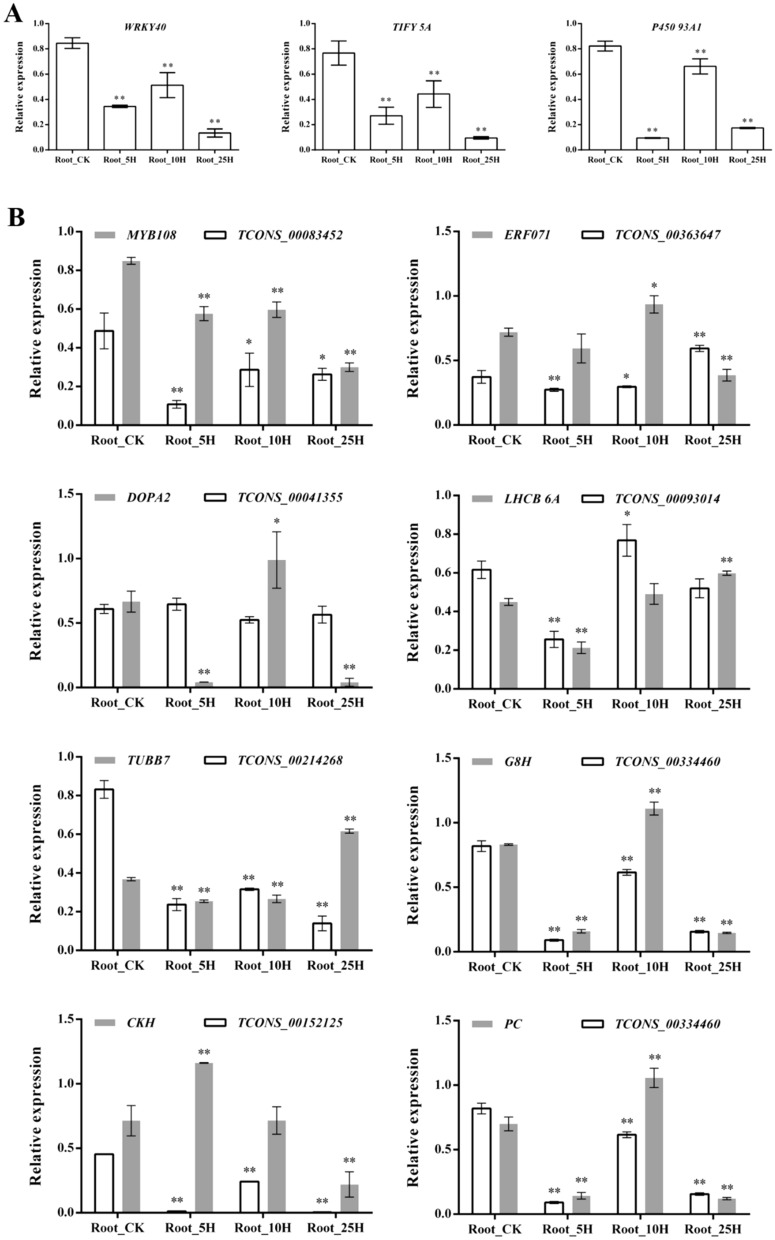
Quantitative real time PCR results of selected differentially expressed genes (DEGs), and long noncoding RNAs (DELs) and their predicted target genes (DETs). (**A**) expression of DEGs without corresponding lncRNAs; (**B**) expression of DELs and their corresponding DETs. *P450 93A1*: *Cytochrome P450 93A1*, *ERF071*: *Ethylene-responsive transcription factor ERF071*, *DOPA2*: *Tyrosine/DOPA decarboxylase 2*; *LHCB6A*: *light harvesting chlorophyll a–b binding protein 6A*, *TUBB7*: *tubulin beta-7 chain like gene*, *G8H*: *geraniol 8-hydroxylase-like*, *CTH*: *cytokinin hydroxylase-like*, *PC*: *Plastocyanin*. Columns marked with ‘*’ or ‘**’ indicate significant or very significant difference with the Root_CK group, respectively.

GO enrichment analysis of DEGs revealed that the ‘oxidation–reduction process’, ‘oxidoreductase activity’, ‘metal ion binding’, ‘cation binding’ and other 79 GO terms were significantly enriched (Supplementary Table S5). KEGG analysis showed that many DEGs were involved in metabolic pathways (188 DEGs), biosynthesis of secondary metabolites (112 DEGs), carbon metabolism (35 DEGs), ribosome (33 DEGs), biosynthesis of amino acids (30 DEGs), glycolysis / gluconeogenesis (26 DEGs), plant hormone signal transduction (23 DEGs), phenylpropanoid biosynthesis (22 DEGs), starch and sucrose metabolism (19 DEGs), phenylalanine metabolism (18 DEGs), plant–pathogen interaction (14 DEGs), amino sugar and nucleotide sugar metabolism (15 DEGs), spliceosome (14 DEGs) and so on (Fig. 5, Supplementary Table S6). Moreover, several alpha-linolenic acid (ALA) metabolism related genes, including three *linoleate 9S-lipoxygenases* and two *lipoxygenases*, were identified as DEGs. Several fatty acid (FA) metabolism-related genes, including two *omega-3 fatty acid desaturases* and two *omega-6 fatty acid desaturases*, were also identified as DEGs (Supplementary Table S3). The expression levels of many transcription factors (TFs) genes were significantly influenced by *Foc*TR4 infection. These DE *TFs* included 29 *ERFs*, 18 *zinc finger proteins*, 12 *WRKYs*, 9 *NACs*, 5 *MYBs* and etc. (Supplementary Table S3). Moreover, many DEGs encoding members of P450, peroxidase, TIFY, proline rich protein, ubiquitin related protein and ACC oxidase gene families. Additionally, many plant defense related genes, such as *chitinases*, *germin like proteins*, *disease resistance proteins*, *thaumatin like proteins* and so on (Supplementary Table S3), also account a large percent of DEGs. And twelve photosynthesis-antenna protein genes and twenty six glycolysis/gluconeogenesis related genes were also identified as DEGs. To validate the gene expression obtained through RNA-Seq, six genes, i.e. *WRKY40*, *TIFY 5A*, *P450 93A1*, *MYB108*, *ERF071*, *DOPA2* and *LHCB 6A*, were selected for qRT-PCR verification and got consistent results, which indicate the trueness of our RNA-Seq data (Fig. 6).

### Function analysis of DELs and their target genes (DETs)

GO enrichment analysis of the 78 DELs’ target genes (DETs) revealed that ‘mannosyl-oligosaccharide glucosidase activity’, ‘glucosidase activity’, ‘obsolete ubiquitin thiolesterase activity’, ‘ubiquitinyl hydrolase activity’ and ‘ergosterol biosynthetic process’ were the top five enriched GO terms (Supplementary Tables S7 and S8). Among the 78 DELs, eight DELs were located in chromosome 5, seven DELs each were in chromosome 4 and 9, and only one DEL was located in chromosome 8 (Fig. 2). KEGG enrichment analysis of the predicted DETs showed that, similar to DEGs, these DETs were also mainly involved in metabolic pathways (112 DETs), biosynthesis of secondary metabolites (61 DETs), ribosome (31 DETs), carbon metabolism (19 DETs), biosynthesis of amino acids (16 DETs), glycolysis / gluconeogenesis (8 DETs), plant hormone signal transduction (13 DETs), phenylpropanoid biosynthesis (14 DETs), starch and sucrose metabolism (11 DETs), amino sugar and nucleotide sugar metabolism (8 DETs), spliceosome (10 DETs), plant–pathogen interaction (8 DETs), phenylalanine metabolism (7 DETs) and so on (Supplementary Table S9). Furthermore, there were 22, 16, 13, 12 and 11 DETs involved in protein processing in endoplasmic reticulum, purine metabolism, RNA degradation, ubiquitin mediated proteolysis and oxidative phosphorylation, respectively.

Among the DETs, 48 were found to be also DEGs (Supplementary Table S10). Some DELs and their corresponding DETs showed the opposite expression pattern in response to *Foc*TR4 infection. For example, the *Foc*TR4-induced TCONS_00083452 was predicted to target a *Foc*TR4 suppressed *MYB108* gene at all the three time points, and our qRT-PCR results also showed the same expression pattern. The *Foc*TR4 induced TCONS_00363647 also showed opposite expression pattern with its predicted target *ERF071*. Our qRT-PCR revealed the same expression pattern at 5 hpi and 10 hpi, but an opposite expression pattern was found at 25 hpi. TCONS_00041355 and its predicted target *DOPA2* also showed opposite expression pattern. Consistently, by using qRT-PCR, opposite expression pattern was also found between TCONS_00041355 and *DOPA2* (Fig. 6).

Some DELs and DEGs showed similar expression pattern. For example: TCONS_00093014 and its target gene *LHCB 6A* both showed down-regulated expression pattern in response to *Foc*TR4 infection according to the RNA-Seq data. Our qRT-PCR also revealed the same expression pattern except that the expression of TCONS_00093014 was up-regulated at 10 hpi. The *Foc*TR4 suppressed TCONS_00214268 was predicted to target *TUBB7* that was down-regulated by *Foc*TR4 infection. Our qRT-PCR results showed the same expression pattern at 5 hpi and 10 hpi, but was opposite at 25 hpi. TCONS_00334460 and its predicted target gene *G8H* were both suppressed by *Foc*TR4 infection. And their expression patterns validated using qRT-PCR were the same as that of RNA-seq. TCONS_00152125 and its target gene *CKH* also showed similar down-regulated expression pattern in response to banana wilt pathogen infection. However, the qRT-PCR results only showed the same expression pattern at 25 hpi, opposite at 5 hpi and 10 hpi. TCONS_00334460 and its target gene *PC* showed similar down-regulated expression pattern during the *Foc*TR4 early infection stages. Our qRT-PCR result was similar to the sequencing data except at 10 hpi.

## Discussion

In this study, the mRNA and lncRNA expression changes in banana root in response to *Foc*TR4 infection in the early stages were investigated using lncRNA sequencing. Totally, 78 DELs, 1220 DE known genes and 100 DE novel genes were identified in *Foc*TR4 infected banana roots compared with healthy banana roots. Functional analysis result revealed that, among these DEGs and DETs, many were known *Foc*TR4 responsive genes that have been identified in previous transcriptomic studies, which further confirmed their roles in the banana response to *Foc* infection. Furthermore, many DEGs and DETs were found to be involved in pathways that have not been reported in previous studies, indicating that these genes and lncRNAs also play part in the early banana-*Foc*TR4 interactions.

### LncRNA sequencing analysis identified many reported and new-found Foc responsive genes and pathways during the early FocTR4 infection stages

Similar to previous studies, most DEGs identified in this study, such as genes encoding chitinases, germin like proteins and disease resistance proteins^10–13,50,68^, were reported to be *Foc*TR4 responsive. These DEGs were mainly involved in a series of pathways such as biosynthesis of secondary metabolites, plant–pathogen interaction, plant hormone signal transduction, phenylalanine metabolism, phenylpropanoid biosynthesis and ALA metabolism^11,13^. One interesting finding is the discovery that ALA metabolism pathway is *Foc*TR4 responsive. The ALA metabolism had been confirmed to play roles in plant–pathogen interaction in many plant species including banana^78,79^. In the study of Wang et al.^11^, they identified the significant enrichment of the ALA metabolism related genes. In our present study, ALA metabolism was also identified to be significantly enriched in *Foc*TR4 infected banana roots. Three *linoleate 9S-lipoxygenases* (up-regulated) and two *lipoxygenases* (down-regulated) were identified as DEGs in our study. Lipoxygenases (LOXs) are key enzymes of JA synthesis, which catalyzes the conversion of α-linoleic acid to hydroperoxy-octadecadienoic acid. Our previous study also revealed that the *Foc*TR4 infection could influence the expression of several banana *LOX* genes^80^. Devi et al.^79^ reported that lipoxygenase metabolites of ALA function in improving pigeon pea resistance against *F. udum* infection. Induction of *OsLOX2/5* expression was proposed to be a potential resolution to the rice blast disease^63^. Zhang et al.^81^ found that the silencing of two lncRNAs of cotton could enhance cotton *Verticillium* wilt resistance by increasing the expression of *LOX1* and *LOX2*. ALA is a polyunsaturated fatty acid (PUFA) belonging to omega-3 fatty acids. Recently, FAs have been proved to be the major carbon source that parasitic fungi acquired from host plant^82,83^. In our previous study, we also found that several FA metabolism related genes were targets of *Foc*TR4 responsive miRNAs^69^. Thus, it was deduced that these genes contributed a lot to the banana resistance by influencing the carbon source exchange between banana and *Foc*TR4^69^.

We also identified the enrichment of photosynthesis related genes, and several *LHCBs* as DEGs, some of which were predicted to be target genes of DELs. Photosynthesis related genes down-regulated in leaves and stems were also down-regulated in non-photosynthetic roots as shown in HLB infected citrus root^84^. The function of LHCB in photosynthesis and leaf development of plant has been well recognized. Recent studies also revealed its role in root^85^ and in plant stress response^45^, suggesting that the enrichment of photosynthesis related genes in *Foc*TR4 infected roots might contribute to banana resistance to pathogen infection. Furthermore, we also identified many genes with unknown functions. Many novel genes discovered showed differential expression in FW infected banana root in the early stages, their functions in the response of banana to *Foc*TR4 infection are therefore need to be further studied.

### LncRNA participate in the banana-FocTR4 interactions by regulating the expression of many known FocTR4- and stress-responsive genes

In Total, we obtained 12,109 lncRNAs in *Foc*TR4 treated and control banana roots. By differential expression analysis, we identified 78 differentially expressed (DE) lncRNAs. GO and KEGG enrichment analysis of the predicted *cis* target genes of DE lncRNAs revealed the enrichments of some reported *Foc*-responsive pathways^10–13^, such as biosynthesis of secondary metabolites, phenylpropanoid biosynthesis, plant–pathogen interaction, plant hormone transduction, and so on. This indicated that the differential expression of these genes were, at least partially, dependent on the regulation of their corresponding lncRNAs.

The expression of genes encoding ACC oxidases and ERFs were reported to be significantly induced by *Foc*1 and *Foc*4^50^. In this study, 29 *ERFs* and 8 *ACC oxidases* were identified as DEGs, and four of these *ERFs* were significantly down-regulated at all the three *Foc*TR4 infected early stages. Additionally, we found that the *Foc*TR4 induced TCONS_00363647 showed opposite expression pattern with its predicted target *ERF071*. Our qRT-PCR result also revealed opposite expression pattern at 25 hpi. It was thus hypothesized that the differential expression of these ethylene signaling related genes play roles in the FW response of banana.

Several *MYB* genes were predicted to be targets of DE lncRNAs. Among them, a *Foc*TR4-suppressed *MYB108* gene was predicted to be target of the *Foc*TR4 induced TCONS_00083452. Our qRT-PCR results revealed the same expression patterns. *MYB108* has been shown to be a defense-related gene in several plants. The cotton *GhMYB108* was reported to be *Verticillium dahlia* inducible. Knockdown of *GhMYB108* increased the susceptibility of cotton plant to *V. dahlia*, while the *GhMYB* over-expression Arabidopsis plants showed enhancement in its tolerance^86^. Moreover, the Arabidopsis *MYB108*, also known as *Botrytis sensitive 1* (*BOS1*), was reported to be required for the plant’s development and biotic and abiotic stress responses^87–89^.

### LncRNA participate in the banana-FocTR4 interactions in the early infection stages by regulating genes involved in some new-found FocTR4-responsive pathways

The enrichment analysis of the DETs also revealed the significant enrichment of some pathways that have not been reported in previous transcriptome studies. These pathways included ribosome, oxidative phosphorylation, lipoic acid metabolism, ubiquitin mediated proteolysis, N-glycan biosynthesis, protein processing in endoplasmic reticulum, DNA damage response and so on.

Li et al.^68^ studied the lncRNA expression pattern in *Foc*TR4 infected ‘Baxijiao’ and ‘Nongke No.1’ at 27 and 51 hpi, and found the enrichment of ribosome and oxidative phosphorylation pathways, which was consistent with our result. The enrichment of ribosome related DETs once again confirmed the translational regulatory role of lncRNAs^52^. The enrichment of oxidative phosphorylation related DETs suggested that lncRNA function in response to *Foc*TR4 infection by regulating this pathway in banana roots.

Lipoic acid, an important natural non-enzymatic antioxidant metabolite, plays roles in protecting cells against oxidative damages^90^. In our present study, we found that several lncRNA target genes were involved in lipoic acid metabolism, and the pathway significantly enriched, suggesting that these lncRNA and their target genes contributed to the ROS scavenging during the early banana-*Foc*TR4 interactions.

Ubiquitin mediated proteolysis has emerged as the one theme in plant–microbe interactions^91^. It is worth mentioning that the ubiquitin-dependent proteasome or ubiquitin/proteasome system (UPR) is involved in the perception and signaling of plant hormones^92^. Consistently, *Foc* infection would influence the phytohormone balance in banana root^69^, suggesting that lncRNA could function in the banana-*Foc*TR4 interactions by regulating ubiquitin mediated proteolysis in response to the phytohormone balance changes caused by pathogen infection.

Endoplasmic reticulum (ER)-based N-glycosylation is emerging as an important participant in plant stress responses^93^. ER is not only responsible for the synthesis and folding of most secretory and transmembrane proteins, but also perceives and responds to cellular biotic and abiotic stresses, i.e., to ER stress^94^. ER stress can be induced by the inhibition of glycosylation, rapid generation of ROS and other biological changes^95^. Our lncRNA data revealed that DETs involved in N-glycan biosynthesis and protein processing in ER were significantly enriched in the *Foc*TR4 infected banana roots during the early infection stages.

*Foc* can secrete toxins to facilitate their infection and growth, which happen to be lethal to banana root cells^96^. In response to stresses, plant cells initiate a series of stress response pathways that are collectively termed as DNA damage response (DDR)^97^. DDR is essential for the detecting and repairing DNA damages caused by intrinsic and extrinsic stresses and play roles in safeguarding the genome and plant survival. The enrichment of the DDR related DETs indicated that lncRNAs may contribute in safeguarding the banana genome and survival of banana root cells against the damages caused by the pathogen.

## Conclusion

In the present study, lncRNA sequencing was used for the investigation of the mRNA and lncRNA expression changes in banana roots at the early *Foc*TR4 infection stages. Consistent with the previous transcriptome studies on banana–*Foc* interactions, many reported *Foc*-responsive DEGs and pathways were also identified in our study. Noteworthily, many of these common DEGs were identified to be targets of DELs, suggesting that lncRNAs participate in the FW responses of banana by regulating these *Foc*-responsive genes. Meanwhile, lncRNAs were also found to serve the functions of regulating ribosome, oxidative phosphorylation, lipoic acid metabolism, N-glycan biosynthesis, protein processing in ER, and DDR related genes in response to *Foc*TR4 infection at the early stages. The results obtained in this study will be helpful in the understanding of the *Foc*TR4-induced transcriptome changes in the banana root and in clarifying the regulatory roles of lncRNAs during banana–*Foc*TR4 interactions.

## Supplementary Information

Supplementary Tables.

## Abbreviations

*Foc*TR4: *Fusarium oxysporum* F. sp. *cubense* Tropical Race 4
FW: Fusarium wilt
lncRNA: Long noncoding RNA
DEG: Differentially expressed gene
DEL: Differentially expressed lncRNA
DETs: Target gene of DEL
bp: Base pair
ERF: Ethylene-responsive transcription factor
DOPA2: Tyrosine/DOPA decarboxylase 2
LHCB: Light harvesting chlorophyll a–b binding protein
TUBB7: Tubulin beta-7 chain like gene
G8H: Geraniol 8-hydroxylase-like
CTH: Cytokinin hydroxylase-like
PC: Plastocyanin
ALA: Alpha-linolenic acid
TF: Transcription factor
FC: Fold change
ER: Endoplasmic reticulum
DDR: DNA damage response
PUFA: Polyunsaturated fatty acid
FA: Fatty acid
LnA: α-Linoleic acid
HPODE: Hydroperoxy-octadecadienoic acid
PCR: Polymerase chain reaction
qRT-PCR: Quantitative real time PCR
UMP: Ubiquitin mediated proteolysis
